# Omicron escapes the majority of existing SARS-CoV-2 neutralizing antibodies

**DOI:** 10.1038/s41586-021-04385-3

**Published:** 2021-12-23

**Authors:** Yunlong Cao, Jing Wang, Fanchong Jian, Tianhe Xiao, Weiliang Song, Ayijiang Yisimayi, Weijin Huang, Qianqian Li, Peng Wang, Ran An, Jing Wang, Yao Wang, Xiao Niu, Sijie Yang, Hui Liang, Haiyan Sun, Tao Li, Yuanling Yu, Qianqian Cui, Shuo Liu, Xiaodong Yang, Shuo Du, Zhiying Zhang, Xiaohua Hao, Fei Shao, Ronghua Jin, Xiangxi Wang, Junyu Xiao, Youchun Wang, Xiaoliang Sunney Xie

**Affiliations:** 1 Biomedical Pioneering Innovation Center (BIOPIC), Peking University, Beijing, P. R. China; 2 Beijing Advanced Innovation Center for Genomics (ICG), Peking University, Beijing, P. R. China; 3 School of Life Sciences, Peking University, Beijing, P. R. China; 4 College of Chemistry and Molecular Engineering, Peking University, Beijing, P. R. China; 5 Joint Graduate Program of Peking–Tsinghua–NIBS, Academy for Advanced Interdisciplinary Studies, Peking University, Beijing, P. R. China; 6 Division of HIV/AIDS and Sex-transmitted Virus Vaccines, Institute for Biological Product Control, National Institutes for Food and Drug Control (NIFDC), Beijing, P. R. China; 7 Tsinghua-Peking Center for Life Sciences, Beijing, P. R. China; 8 Beijing YouAn Hospital, Capital Medical University, Beijing, P. R. China; 9 Beijing Ditan Hospital, Capital Medical University, Beijing, P. R. China; 10 CAS Key Laboratory of Infection and Immunity, National Laboratory of Macromolecules, Institute of Biophysics, Chinese Academy of Sciences, Beijing, P. R. China

**Keywords:** Infectious diseases, High-throughput screening, SARS-CoV-2

## Abstract

The SARS-CoV-2 B.1.1.529 (Omicron) variant contains 15 mutations of the receptor-binding domain (RBD). How Omicron evades RBD-targeted neutralizing antibodies requires immediate investigation. Here we use high-throughput yeast display screening^1,2^ to determine the profiles of RBD escaping mutations for 247 human anti-RBD neutralizing antibodies and show that the neutralizing antibodies can be classified by unsupervised clustering into six epitope groups (A–F)—a grouping that is highly concordant with knowledge-based structural classifications^3–5^. Various single mutations of Omicron can impair neutralizing antibodies of different epitope groups. Specifically, neutralizing antibodies in groups A–D, the epitopes of which overlap with the ACE2-binding motif, are largely escaped by K417N, G446S, E484A and Q493R. Antibodies in group E (for example, S309)^6^ and group F (for example, CR3022)^7^, which often exhibit broad sarbecovirus neutralizing activity, are less affected by Omicron, but a subset of neutralizing antibodies are still escaped by G339D, N440K and S371L. Furthermore, Omicron pseudovirus neutralization showed that neutralizing antibodies that sustained single mutations could also be escaped, owing to multiple synergetic mutations on their epitopes. In total, over 85% of the tested neutralizing antibodies were escaped by Omicron. With regard to neutralizing-antibody-based drugs, the neutralization potency of LY-CoV016, LY-CoV555, REGN10933, REGN10987, AZD1061, AZD8895 and BRII-196 was greatly undermined by Omicron, whereas VIR-7831 and DXP-604 still functioned at a reduced efficacy. Together, our data suggest that infection with Omicron would result in considerable humoral immune evasion, and that neutralizing antibodies targeting the sarbecovirus conserved region will remain most effective. Our results inform the development of antibody-based drugs and vaccines against Omicron and future variants.

## Abstract (web summary)

A high-throughput yeast display platform is used to analyse the profiles of mutations in the SARS-CoV-2 receptor-binding domain (RBD) that enable escape from antibodies, and suggests that most anti-RBD antibodies can be escaped by the Omicron variant.

## Main

**Extended Data Fig. 1:**
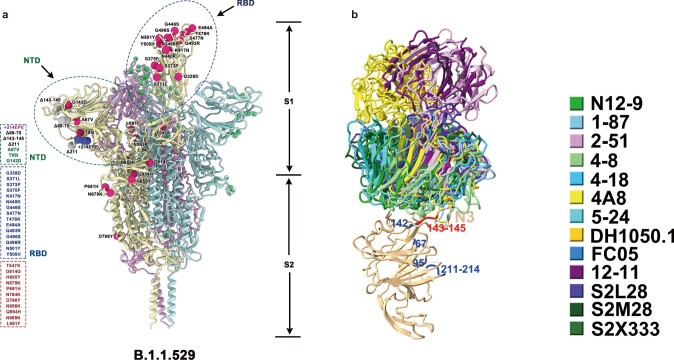
Illustration of the SARS-CoV-2 spike protein with Omicron’s mutations. **a**, SARS-CoV-2 D614G spike protein structure overlayed with Omicron mutations. Omicron’s (BA.1) popular mutations are marked by red (for substitutions), blue (for insertions) and grey balls (for deletions). **b**, NTD-binding neutralizing antibodies shown together in complex with NTD. Substitutions and deletions of Omicron NTD are coloured blue and red, respectively.

The SARS-CoV-2 variant B.1.1.529 was first reported to the World Health Organization (WHO) on 24 November 2021. It spread rapidly, and the WHO classified it as a variant of concern only two days after, designating it as Omicron^8,9^. An unusually large number of mutations are found in Omicron, including more than 30 in the spike protein (Extended Data Fig. 1a). The RBD, which is responsible for interacting with the angiotensin-converting enzyme 2 (ACE2) receptor, contains 15 of these mutations: G339D, S371L, S373P, S375F, K417N, N440K, G446S, S477N, T478K, E484A, Q493R, G496S, Q498R, N501Y and Y505H. Some of these mutations are very concerning because of their well-understood functional consequences. For example, K417N and N501Y contribute to immune escape and higher infectivity^10–13^.The functional effects of many other mutations still require investigation.

The spike protein is the target of essentially all neutralizing antibodies that are found in the sera of convalescent individuals or that are elicited by vaccines. Most of the N-terminal domain (NTD)-directed neutralizing antibodies target an antigenic ‘supersite’ in the NTD, which involves the N3 (residues 141–156) and N5 (residues 246–260) loops^14,15^; these antibodies are thus very susceptible to NTD mutations. Omicron carries the Δ143–145 mutation, which would alter the N3 loop and is likely to result in the immune escape of most anti-NTD neutralizing antibodies (Extended Data Fig. 1b). Compared to NTD-targeting neutralizing antibodies, RBD-targeting neutralizing antibodies are particularly abundant and potent, and display diverse epitopes. An evaluation of how Omicron affects the neutralization capability of anti-RBD neutralizing antibodies of diverse classes and epitopes is urgently needed.

**Fig. 1:**
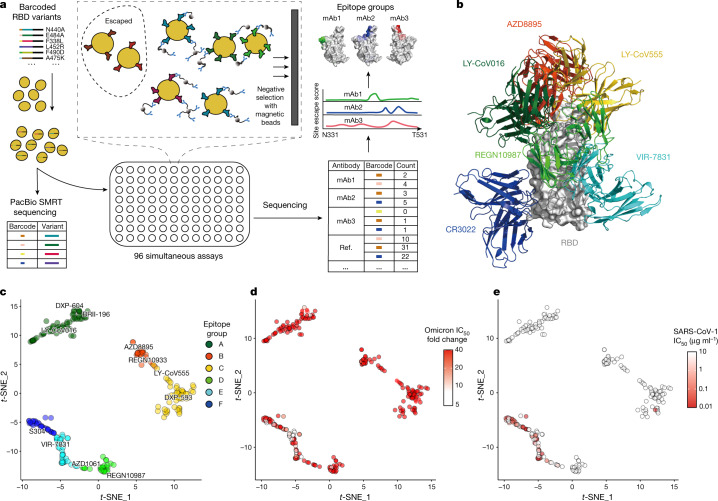
Omicron greatly reduces the neutralization potency of neutralizing antibodies of diverse epitopes. **a**, Schematic of MACS-based high-throughput yeast display mutation screening. mAb, monoclonal antibody. **b**, Representative antibody structures of each epitope group. **c**, *t*-distributed stochastic neighbour embedding (*t*-SNE) and unsupervised clustering of SARS-CoV-2 human neutralizing antibodies on the basis of each antibody escaping mutation profile. A total of six epitope groups (groups A–F) could be defined. **d**, Neutralization of the Omicron variant (spike-pseudotyped VSV) by 247 RBD neutralizing antibodies. Shades of red show the fold change in IC_50_ compared with D614G for each antibody. **e**, Neutralization of SARS-CoV-1 (spike-pseudotyped VSV) by 247 RBD neutralizing antibodies. Shades of red show the IC_50_ value (μg ml^−1^) of each antibody. All pseudovirus neutralization assays were conducted in biological duplicates or triplicates.

**Extended Data Fig. 2:**
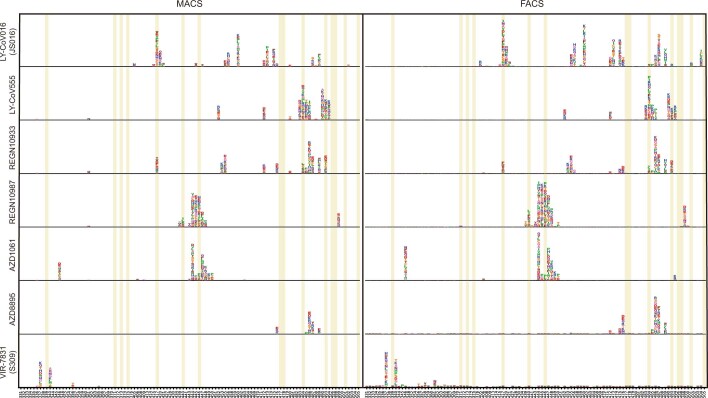
Comparison between FACS- and MACS-based deep mutational scanning. Deep mutational scanning maps with MACS-based (left) and FACS-based assays (right) of seven therapeutic neutralizing antibodies that have received emergency use authorization. Sites mutated in the Omicron variant are highlighted. Mutation amino acids of each site are shown by single letters. The heights represent mutation escape score, and colours represent chemical properties. FACS-based data were obtained from public datasets by Jesse Bloom.

RBD-directed SARS-CoV-2 neutralizing antibodies can be assigned into different classes or binding sites on the basis of structural analyses by cryo-electron microscopy or high-resolution crystallography^3–5^. However, analysis based on structural data only indicates the contacting amino acids, and does not enable the escaping mutations for a specific antibody to be identified. Advances in deep antigen mutation screening using a fluorescence-activated cell sorting (FACS)-based yeast display platform has allowed the quick mapping of all single-amino-acid mutations in the RBD that affect the binding of SARS-CoV-2 RBD neutralizing antibodies^1,16^. The method has proven highly effective in predicting the efficacy of neutralizing-antibody-based drugs towards mutations^2^. However, to study how human humoral immunity may react to highly mutated variants such as Omicron requires mutation profiling of a large collection of neutralizing antibodies that target different regions of the RBD, and mutation screening with the FACS-based yeast display method is limited by low experimental throughput. Here we developed a magnetic-activated cell sorting (MACS)-based screening method that increases the throughput by nearly 100-fold while obtaining a comparable data quality to FACS (Fig 1a, Extended Data Fig. 2). Using this method, we rapidly characterized the profile of RBD escaping mutations for a total of 247 neutralizing antibodies (Supplementary Data 1). Half of the neutralizing antibodies were part of the antibodies identified by us using single-cell V(D)J sequencing of antigen-specific memory B cells from individuals who had been infected with SARS-CoV-2 (hereafter, SARS-CoV-2 convalescent individuals); individuals who had been vaccinated against SARS-CoV-2; and individuals with a previous infection of SARS-CoV-1 (SARS-CoV-1 convalescent individuals) who had recently been vaccinated against SARS-CoV-2 (Supplementary Data 2). The other half of the neutralizing antibodies were identified by groups worldwide^3,5,6,11,17–40^ (Supplementary Table 1).

The high-throughput screening capability allowed us to classify these neutralizing antibodies into six epitope groups (A–F) using unsupervised clustering without dependence on structural studies, and the grouping is highly concordant with knowledge-based structural classifications^3–5^ (Fig. 1b, c). In particular, group A–D neutralizing antibodies largely correspond to the RBS A–D neutralizing antibodies described by Yuan et al.^4^, and overlap with the class 1–2 neutralizing antibodies described by Barnes et al.^3^ in general. The epitopes of these neutralizing antibodies largely overlap with RBD residues that are involved in binding to ACE2. Group A and B neutralizing antibodies, represented by LY-CoV016 and AZD8895, respectively, can usually only bind to the RBDs in the ‘up’ conformation, whereas most of the group C and D antibodies—such as LY-CoV555 and REGN-10987—bind to RBDs regardless of their ‘up’ and ‘down’ conformations. Group E and F neutralizing antibodies are very similar to the class 3 and 4 antibodies described by Barnes et al.^3^, and target the S309 (VIR-7831) site and CR3022 site, which could exhibit pan-sarbecovirus neutralization capacity (Fig 1e). Most of these neutralizing antibodies neutralize SARS-CoV-2 using mechanisms other than directly interfering with ACE2 binding.

**Extended Data Fig. 3:**
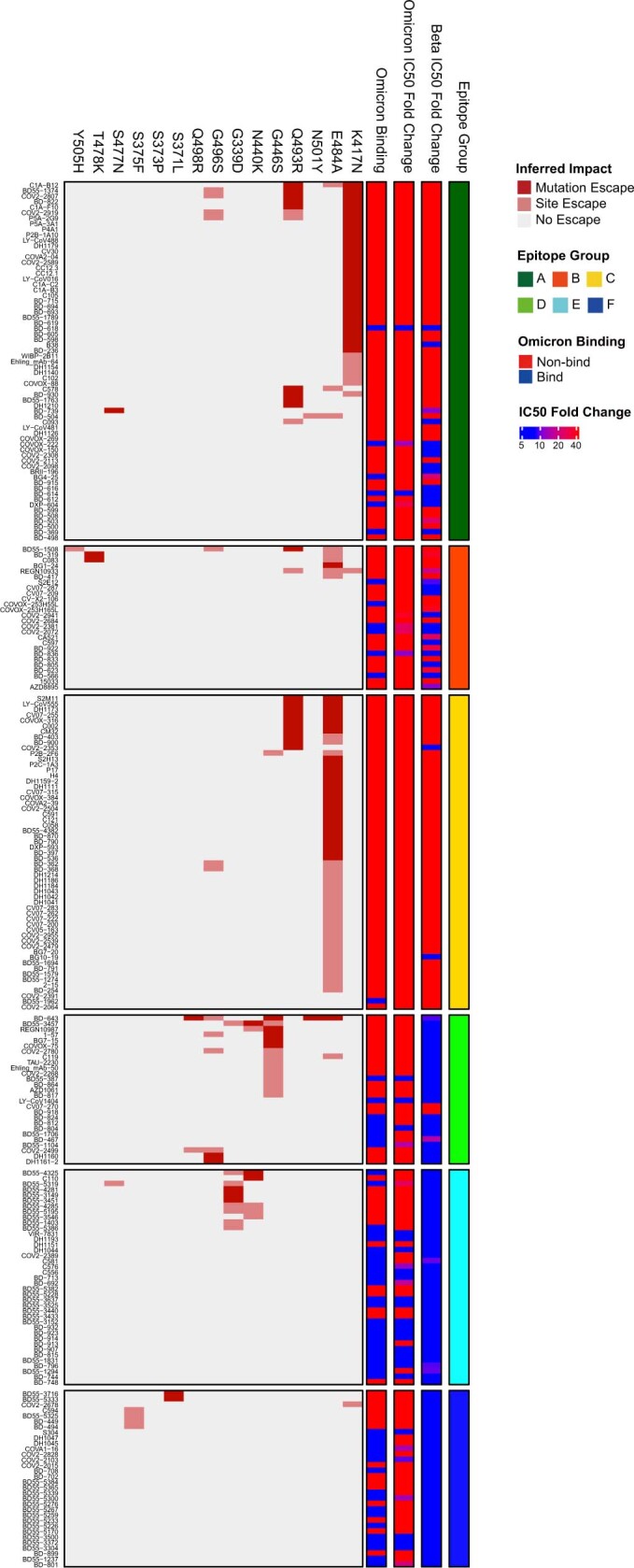
Omicron neutralization IC_50_ fold-change distribution of 247 neutralizing antibodies of diverse epitopes. Fold-change of IC_50_ (VSV pseudovirus neutralization) compared to D614G by Beta and Omicron (BA.1) are shown for all 247 neutralizing antibodies tested. The effect of each RBD mutation of Omicron on antibody binding is inferred from yeast display mutation screening. Each antibody’s binding to Omicron RBD was validated through ELISA. All neutralization and ELISA assays were conducted in biological duplicates.

Inferred from the escaping mutation profiles, various single mutations of Omicron could impair neutralizing antibodies of different epitope groups (Extended Data Fig. 3). Specifically, neutralizing antibodies in groups A–D, the epitopes of which overlap with the ACE2-binding motif, are largely escaped by the single mutations K417N, G446S, E484A, and Q493R. In addition, a subset of neutralizing antibodies of groups E and F are escaped by single mutations of G339D, N440K, S371L and S375F. However, owing to the extensive mutations accumulated on the RBD of Omicron, studying the response of neutralizing antibodies to Omicron only in the context of single mutations is insufficient. Indeed, Omicron pseudovirus neutralization and spike protein enzyme-linked immunosorbent assay (ELISA) showed that neutralizing antibodies that tolerate single mutations could also be escaped by Omicron owing to multiple synergetic mutations on their epitopes (Fig 1d, Extended Data Fig. 3). In total, over 85% of the tested human neutralizing antibodies are escaped, suggesting that Omicron could cause substantial humoral immune evasion and potential antigenic shifting.

**Fig. 2:**
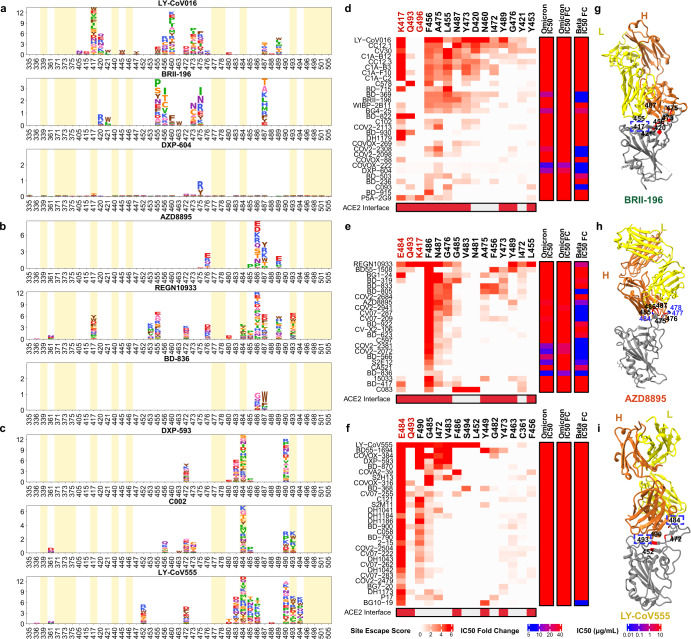
The neutralizing abilities of group A–C antibodies are mostly abolished by Omicron. **a**–**c**, Escaping mutation profiles of representative neutralizing antibodies for group A (**a**), B (**b**) and C (**c**). For each site, the height of a letter indicates the detected mutation escape score of its corresponding residue. Sites mutated in Omicron are highlighted. **d**–**f**, Heat maps of site escape scores for neutralizing antibodies of epitope group A (**d**), B (**e**) and C (**f**). ACE2 interface residues are annotated with red blocks, and mutated sites in Omicron are marked in red. Annotations on the right side of heat maps represent the pseudovirus neutralizing IC_50_ fold change (FC) for Omicron and Beta compared to D614G. **g**–**i**, Representative structures of group A (**g**), group B (**h**) and group C (**i**) antibodies in complex with the RBD. Residues that are involved in important contacts are labelled. Omicron mutations are marked in blue. Antibody escaping mutations (Omicron) inferred from yeast display are labelled with squares.

**Extended Data Fig. 4:**
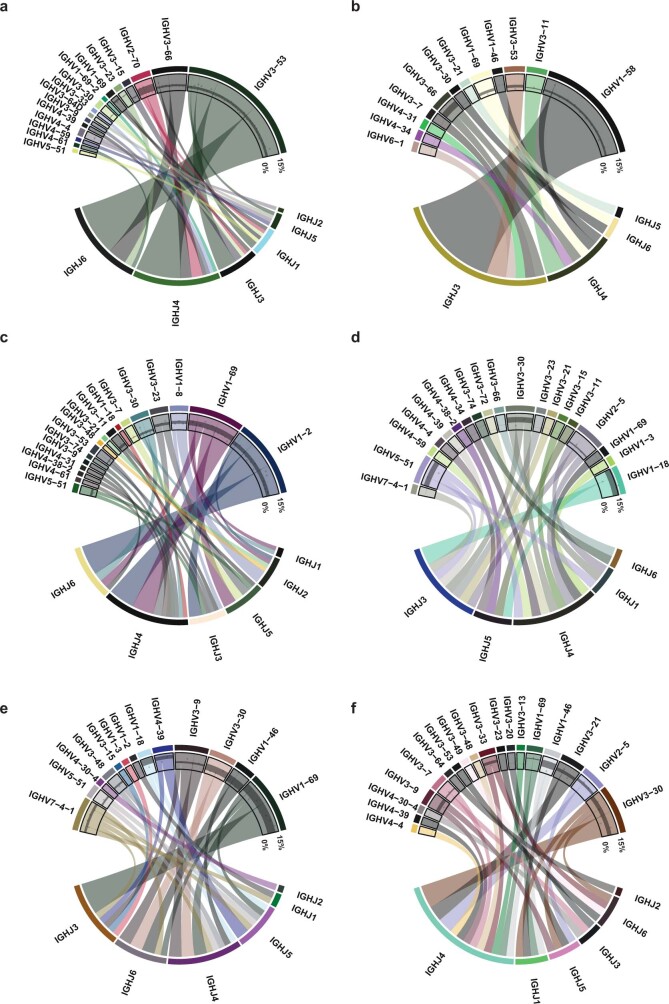
Heavy chain V/J segment recombination of neutralizing antibodies of each epitope group. **a**–**f**, Chord diagrams showing the heavy chain V segment and J segment recombination of epitope group A (**a**), B (**b**), C (**c**), D (**d**), E (**e**) and F (**f**). The width of the arc linking a V segment to a J segment indicates the antibody number of the corresponding recombination. The inner layer scatter plots show the V segment amino acid mutation rate, and black strips show the 25%~75% quantile of mutation rates.

**Extended Data Fig. 5:**
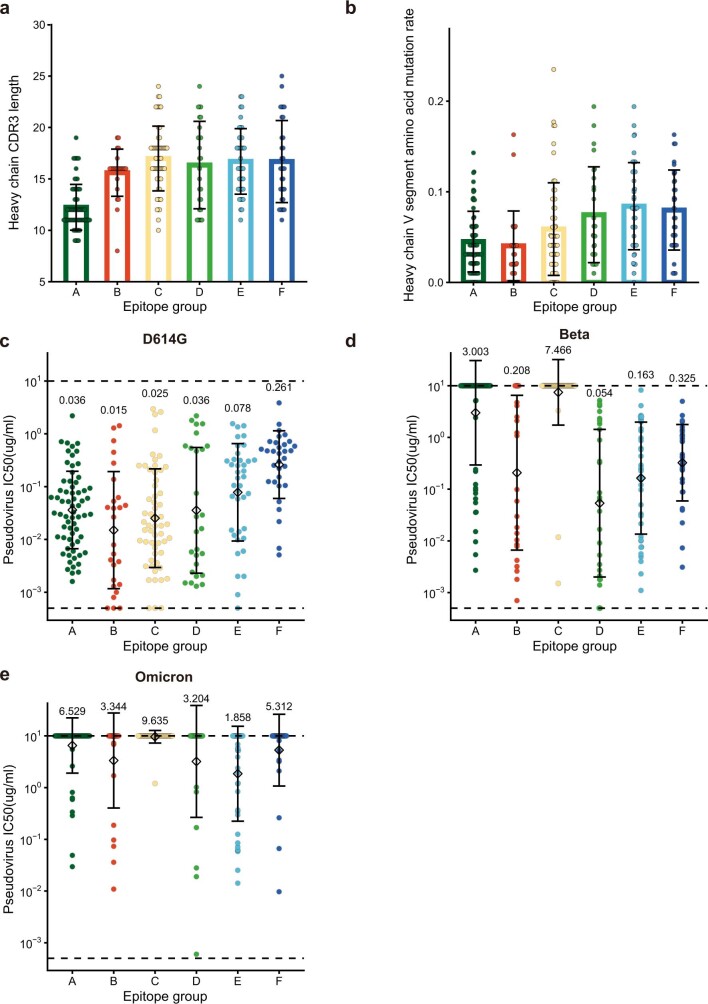
Neutralization potency, heavy chain CDR3 length and mutation rate distribution for neutralizing antibodies of each epitope group. **a**, The length of H chain complementarity-determining region 3 (HCDR3) amino acid sequence for neutralizing antibodies in each epitope group (n = 66, 26, 57, 27, 39, 32 antibodies for epitope group A, B, C, D, E, F, respectively). HCDR3 lengths are displayed as mean  ± s.d. **b**, The V segment amino acid mutation rate for neutralizing antibodies in each epitope group (n = 66, 26, 57, 27, 39, 32 antibodies for epitope group A, B, C, D, E, F, respectively). Mutation rates are calculated are displayed as mean ± s.d. **c**–**e**, The IC_50_ against D614G (**c**), Beta (**d**) and Omicron (**e**) variants for neutralizing antibodies in each epitope group (n = 66, 26, 57, 27, 39, 32 antibodies for epitope group A, B, C, D, E, F, respectively). IC_50_ values are displayed as mean  ±  s.d. in the log10 scale. Pseudovirus assays for each variant are biologically replicated twice. Dotted lines show the detection limit, which is from 0.0005 μg/mL to 10 μg/mL. IC_50_ geometric means are also labelled on the figure.

**Extended Data Fig. 6:**
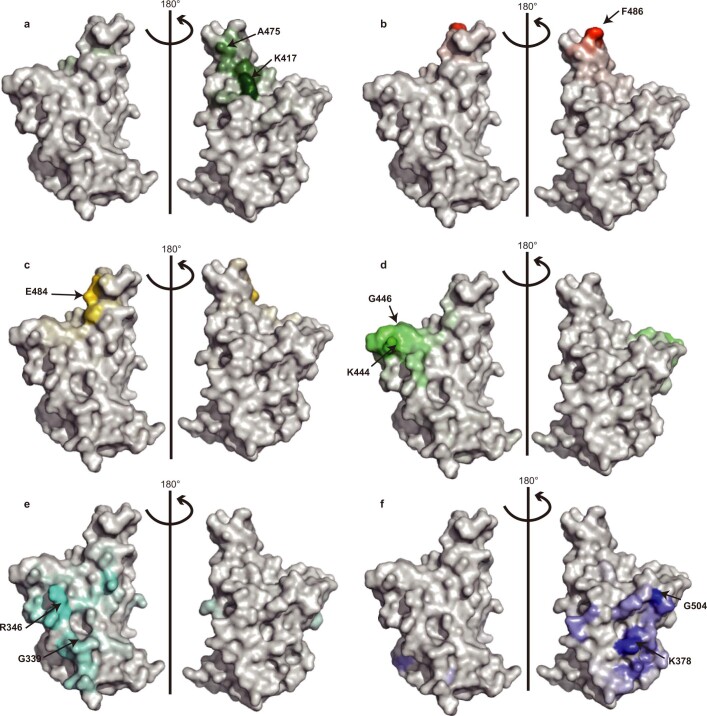
Escape hotspots of different epitope groups on the RBD surface. **a**–**f**, Aggregated site escape scores of antibodies for epitope group A–F, respectively. Epitope groups are distinguished by distinct colours, and the shades show normalized site escape scores. Escape hotspots of each epitope group are annotated by arrows.

**Extended Data Fig. 7:**
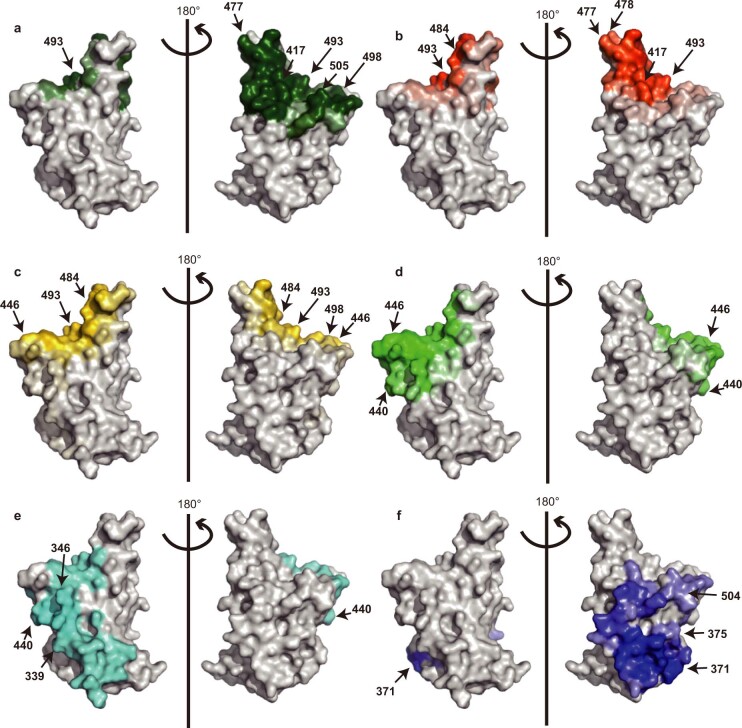
Antibody-RBD interface distribution for neutralizing antibodies of each epitope group. **a**–**f**, Aggregated antibody-antigen interface of antibodies for epitope group A-F, respectively. Antibody-antigen interface was indicated from publicly available structures of neutralizing antibodies in complex with SARS-CoV-2 RBD. Different colours distinguish epitope groups, and the shade reflects group-specific site popularity to appear on the complex interface. Shared interface residues (Omicron) of each group are annotated.

**Extended Data Fig. 8:**
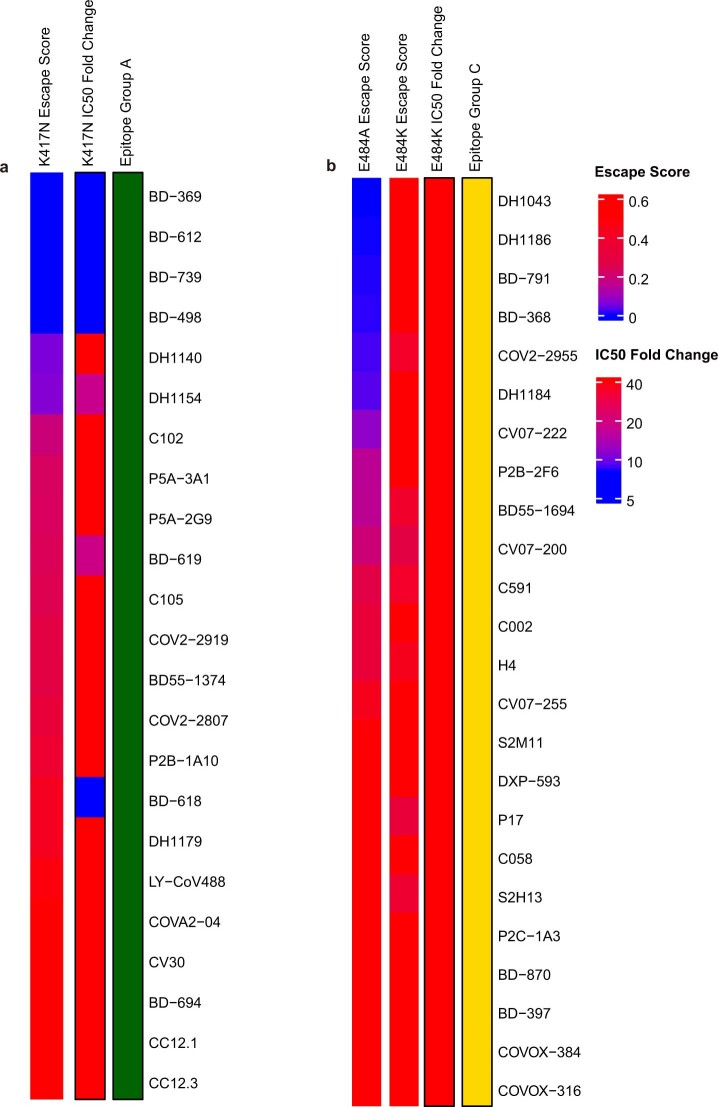
Comparison between mutation escape scores estimated from yeast display and neutralization of variants carrying corresponding mutations. **a**, K417N escape scores and corresponding K417N pseudovirus neutralizing IC_50_ fold change compared to D614G pseudovirus of antibodies within epitope group A. **b**, E484K/E484A escape scores and corresponding E484K pseudovirus neutralizing IC_50_ fold change compared to D614G pseudovirus of antibodies within epitope group C.

It is crucial to analyse how each group of neutralizing antibodies reacts to Omicron to inform the development of drugs and vaccines that are based on these antibodies. Group A neutralizing antibodies mainly comprise antibodies that are encoded by the *VH3-53* and *VH3-66* (also known as *IGHV3-53* and *IGHV3-66*) germline genes; these are present at high levels in our present collection of SARS-CoV-2 neutralizing antibodies^17,21,22,26,41–43^, including several antibodies that have obtained emergency use authorization (CB6/LY-CoV016)^19^ or that are currently being studied in clinical trials (P2C-1F11/BRII-196 and BD-604/DXP-604)^18,44^ (Fig. 2a, Extended Data Fig. 4a). Group A neutralizing antibodies often exhibit fewer somatic mutations and have a shorter complementarity-determining region 3 (CDR3) length compared to other groups (Extended Data Fig. 5a, b). The epitopes of these antibodies extensively overlap with the binding site of ACE2 and are frequently evaded by RBD mutations at the K417, D420, F456, A475 and L455 sites (Fig 2d, Extended Data Figs. 6a, 7a). Most neutralizing antibodies in group A were already escaped by the B.1.351 (Beta) variant (Extended Data Fig. 5d); specifically, by the K417N mutation (Extended Data Fig. 8a), owing to a critical salt-bridge interaction between Lys417 and a negatively charged residue in the antibody (Fig. 2g). Neutralizing antibodies that survived the Beta strain, such as BRII-196 and DXP-604, are insensitive to the K417N single-site change but could also be heavily affected by the combination of K417N and other RBD mutations located on their epitopes, such as S477N, Q493R, G496S, Q498R, N501Y and Y505H of Omicron, thus causing a loss or reduction of neutralization (Fig 2d, Extended Data Fig. 7a).

The neutralizing antibodies encoded by *VH1-58* (*IGHV1-58*) are enriched in group B (Extended Data Fig. 4b). These antibodies—for example, AZD8895 (ref. ^36^), REGN-10933 (ref. ^42^) and BD-836 (ref. ^45^)—bind to the left shoulder of the RBD, often focusing on the far tip (Fig. 2h). These neutralizing antibodies are very sensitive to the changes at the F486, N487 and G476 sites (Fig 2b, Extended Data Fig. 6b). However, F486 and a few other major targeting sites of these neutralizing antibodies are critically involved in ACE2 binding, and therefore they are generally more difficult to escape. A subset of neutralizing antibodies in group B, such as AZD8895 and BD-836, could survive the Beta variant (Fig 2e); however, Omicron significantly reduced the binding affinity of group B neutralizing antibodies to the RBD, potentially as a result of S477N/T478K/E484A on their epitope^46^ (Extended Data Fig. 7b), resulting in the loss of neutralization.

Group C neutralizing antibodies are frequently encoded by *VH1-2* and *VH1-69* (*IGHV1-2* and *IGHV1-69*) (Extended Data Fig. 4c). Most antibodies in this group could bind to both ‘up’ and ‘down’ RBDs, resulting in higher neutralization potency compared to other groups (Fig. 2c, Extended Data Fig. 5c). Several highly potent antibodies are found in group C, including BD-368-2/DXP-593 (ref. ^44^), C002 (ref. ^3^) and LY-CoV555 (ref. ^47^). They bind to the right shoulder of the RBD (Fig. 2i), and are mostly susceptible to changes at E484 (Extended Data Figs. 6c, 7c), such as the E484K mutation found in Beta (Fig. 2f). The E484A mutation that is seen in Omicron elicited a similar escaping effect, although the change to alanine is slightly subtler, and could be tolerated by certain antibodies in this group (Extended Data Fig. 8b). All group C neutralizing antibodies tested are escaped by Omicron.

**Fig. 3:**
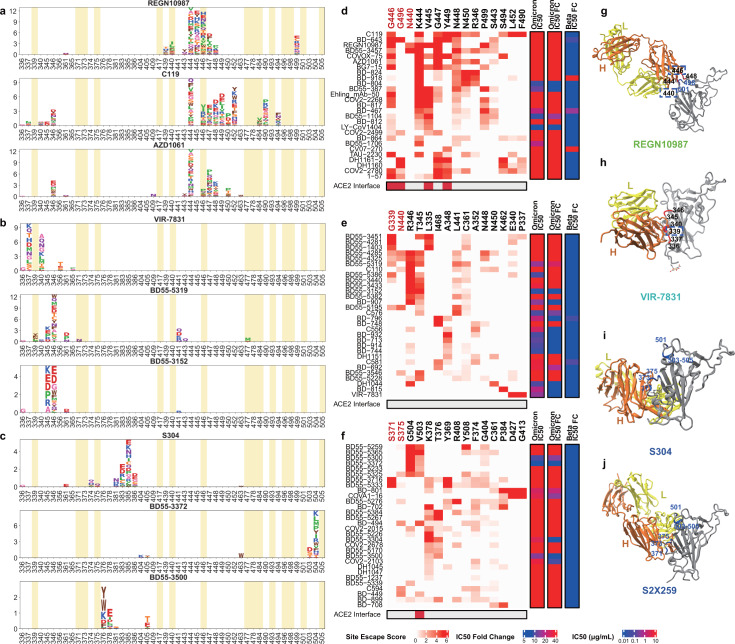
Most group D and E neutralizing antibodies are escaped by Omicron. **a**–**c**, Escaping mutation profiles of representative neutralizing antibodies for groups D (**a**), E (**b**) and F (**c**). For each site, the height of a letter indicates the detected mutation escape score of its corresponding residue. Sites mutated in Omicron are highlighted. **d**–**f**, Heat maps of site escape scores for neutralizing antibodies of epitope groups D (**d**), E (**e**) and F (**f**). ACE2 interface residues are annotated with red blocks, and mutated sites in Omicron are marked in red. Annotations on the right side of heat maps represent the pseudovirus neutralizing IC_50_ fold change (FC) for Omicron and Beta compared to D614G. **g**–**j**, Representative structures of group D (**g**), E (**h**) and F (**i**, **j**) antibodies in complex with the RBD. Residues that are involved in important contacts are labelled. Omicron mutations are marked in blue. Antibody escaping mutations (Omicron) inferred from yeast display are labelled with squares.

Group D neutralizing antibodies consist of diverse IGHV gene-encoded antibodies (Extended Data Fig. 4d). Prominent members in this group include REGN-10987 (ref. ^42^) and AZD1061 (ref. ^36^) (Fig. 3a). They further rotate down from the RBD right shoulder towards the S309 site when compared to group C antibodies (Fig. 3g). As a loop formed by residues 440–449 in the RBD is critical for the targeting of this group of antibodies, they are sensitive to changes at N440, K444, G446 and N448 (Extended Data Figs. 6d, 7d). Most neutralizing antibodies in group D remain active against Beta; however, G446S would substantially affect their neutralization capability against Omicron (Fig. 3d). Also, for those antibodies that could tolerate a G446S single mutation, the N440K/G446S combination may considerably reduce their binding affinity, with the result that most group D antibodies are escaped by Omicron.

Group E and F neutralizing antibodies are rarer when compared to the other four groups. The archetypal member of each group was originally isolated from a SARS-CoV-1 convalescent individual, and exhibits SARS-CoV-2 cross-neutralizing activity. There is no clear V(D)J convergent effect compared to groups A, B and C (Extended Data Fig. 4e, f), and the mutation rate and CDR3 length are larger than other groups. Neutralizing antibodies in groups E and F rarely compete with ACE2; thus, their average half-maximal inhibitory concentration (IC_50_) is higher than that of antibodies in groups A–D (Extended Data Fig. 5c). Neutralizing antibodies in group E—such as VIR-7831/S309—may recognize a mixed protein and carbohydrate epitope that involves the *N*-linked glycan on N343 (ref. ^6^) (Fig. 3h). Inferred from the escaping mutation profiles (Fig. 3b), group E antibodies are often sensitive to changes at G339, T345 and R346 (Extended Data Figs. 6e, 7e). The G339D mutation would affect the neutralization performance of a subset of neutralizing antibodies (Fig. 3e). Also, part of the epitope of group E antibodies would extend to the 440–449 loop, rendering them sensitive to the N440K mutation in Omicron (Fig. 3e). Notably, the frequency of Omicron with the R346K mutation is continuously increasing, which may severely affect the neutralization capacity of group E antibodies.

**Fig. 4:**
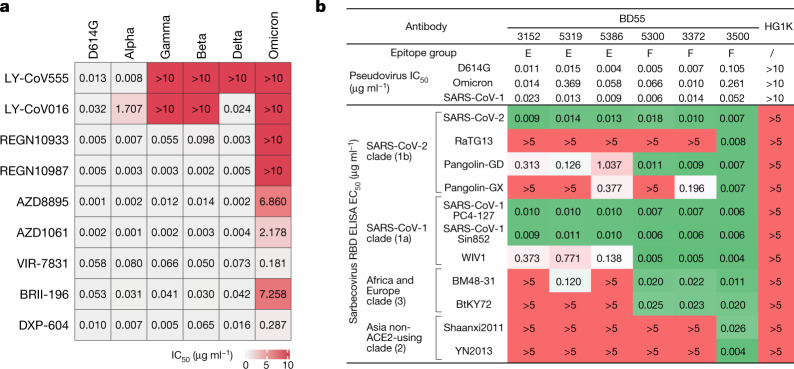
Omicron escapes most neutralizing-antibody-based drugs. **a**, Neutralization of SARS-CoV-2 variants of concern (pseudotyped VSV) by nine neutralizing-antibody-based drugs. The pseudovirus neutralization assays for every VOC were performed in biological triplicates. The IC_50_ values shown are the average of three replicates shown in Extended Data Fig. 9. **b**, The sarbecovirus neutralization and binding capability (half-maximal effective concentration, EC_50_) of selected potent Omicron-neutralizing antibodies. The monoclonal antibody HG1K (IgG1 antibody against influenza A virus subtype H7N9) was used as the negative control.

Group F neutralizing antibodies (for example, S304) target a cryptic site in the RBD that is generally not exposed (Fig. 3i), and therefore their neutralizing activities are generally weaker^7^. Group F antibodies are often sensitive to changes at F374, T376 and K378 (Extended Data Figs. 6f, 7f). A loop involving the RBD residues 371–375 lies in the ridge between the E and F sites; thus, a subset of group F antibodies—including some group E antibodies—could be affected by the S371L/S373P/S375F mutations if their epitopes extend to this region (Fig. 3c, f). Of note, some group F antibodies are highly sensitive to V503 and G504, similar to the epitopes of S2X259 (Fig. 3f, j), suggesting that they can compete with ACE2. Indeed, several neutralizing antibodies, such as BD55-5300 and BD55-3372, exhibit higher neutralization potency than other antibodies in group F (Figs. 3c, 4b). However, the neutralization capability of these antibodies might be undermined by N501Y and Y505H in Omicron (Fig. 3j).

**Extended Data Fig. 9:**
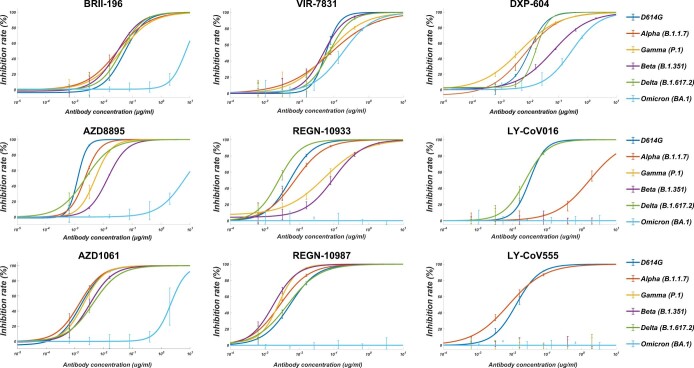
Pseudovirus neutralization of neutralizing-antibody-based drugs against SARS-CoV-2 variants of concern. Pseudovirus (VSV-based) assays were performed using Huh-7 cells. Data are collected from three biological replicates and represented as mean ± s.d.

**Extended Data Fig. 10:**
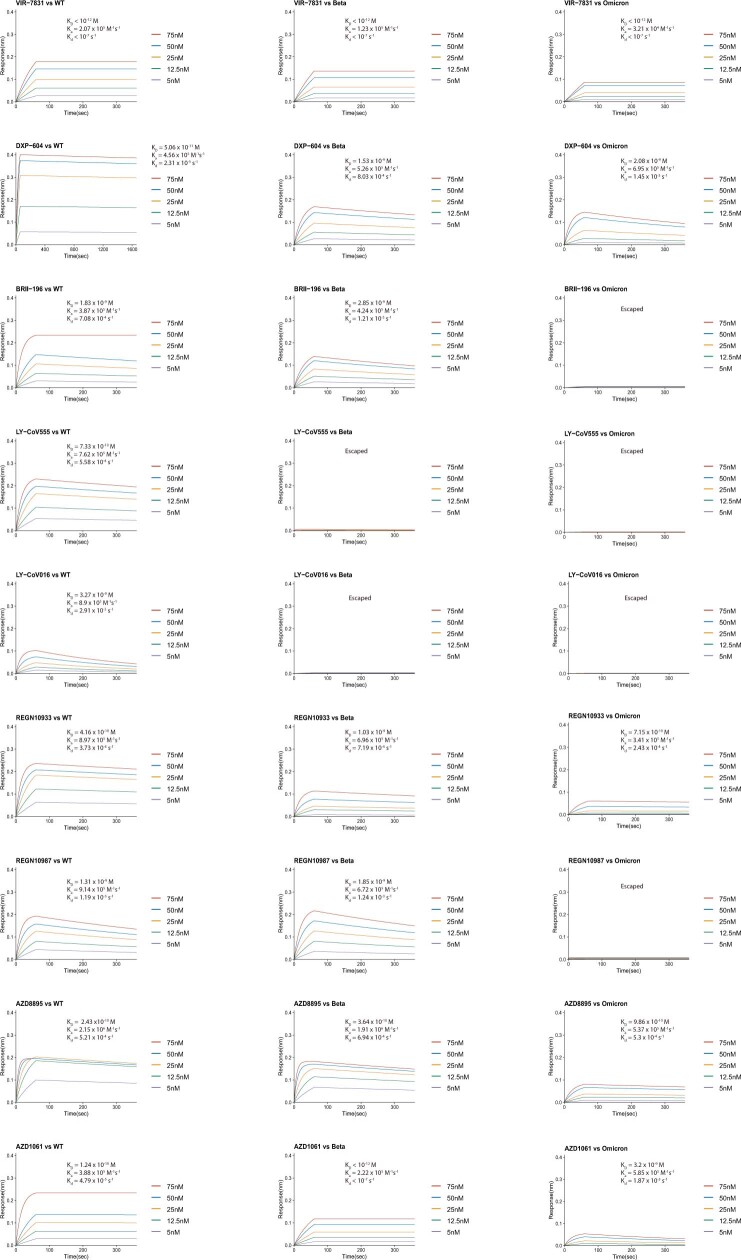
BLI response between neutralizing-antibody-based drugs and the RBD of SARS-CoV-2 wild type, Beta or Omicron strains. Antibodies were captured by Protein A sensor. The concentrations of RBD are shown in different colours. Dissociation constant (*K*_D_), association constant (*k*_a_), and dissociation rate constant (*k*_d_) are labelled. Neutralizing antibodies without binding are marked as ‘Escaped’.

With regard to drugs based on neutralizing antibodies, consistent with their escaping mutation profiles, the neutralization potency of LY-CoV016, LY-CoV555, REGN-10933, REGN-10987 and AZD1061 are greatly reduced by Omicron (Fig. 4a, Extended Data Fig. 9). The binding affinities of AZD8895 and BRII-196 towards the Omicron RBD are also largely reduced, probably owing to multiple mutations accumulating on the epitopes of these antibodies, such that AZD8895 and BRII-196 did not neutralize Omicron (Extended Data Fig. 10). BRII-198 was not tested as the antibody sequence was not released. VIR-7831 retains strong RBD-binding capability; although G339 is part of its epitope, the G339D mutation in Omicron does not appear to affect the binding of VIR-7831. However, the IC_50_ of VIR-7831 is reduced to 181 ng ml^−1^, and may be subject to further reduction against Omicron with R346K. The binding affinity of DXP-604 against the Omicron RBD is markedly reduced compared to the wild-type RBD; nonetheless, it can still neutralize Omicron at an IC_50_ of 287 ng ml^−1^—a reduction of nearly 30-fold compared to wild type (Fig. 4a). In addition, several neutralizing antibodies in groups E and F have shown high potency against Omicron and broad pan-sarbecovirus neutralization ability, suggesting that they have promise for the development of neutralizing-antibody-based drugs (Fig. 4b). Many more neutralizing antibodies identified from SARS-CoV-1 convalescent individuals who have been vaccinated are waiting to be characterized.

The high-throughput yeast screening method provides a laboratory means for quickly examining the epitope of a certain neutralizing antibody; however, the throughput that can be achieved using FACS is limited and cannot be used to evaluate a large library of antibodies. Using MACS, we were able to increase the throughput by two orders of magnitude. In doing so, we were able to gain statistical confidence for the survival proportion of anti-RBD neutralizing antibodies in each epitope group against Omicron. The experimental accuracy for predicting the neutralization reduction for single-amino-acid mutations is relatively high (Extended Data Fig. 8a, b); however, mutation screening through yeast display is not at present able to effectively examine the consequences of multiple mutations simultaneously, and this will require further technical optimization.

So far, a large number of SARS-CoV-2 anti-RBD neutralizing antibodies have been identified from SARS-CoV-2 convalescent individuals and from individuals who have been vaccinated. The most potent antibodies are frequently found in groups A–D, which tend to directly interfere with the binding of ACE2. Nevertheless. the neutralizing powers of these antibodies are often abrogated by RBD mutations in the evolutionary arms race between SARS-CoV-2 and human humoral immunity. Indeed, we showed that Omicron would escape most of the SARS-CoV-2 neutralizing antibodies in this collection (Extended Data Fig. 5e). On the other hand, group E and F antibodies are less affected by Omicron, probably because they are not abundant in the population^48^ and hence exert less evolutionary pressure for RBD to mutate in the corresponding epitope groups. These neutralizing antibodies target conserved RBD regions in sarbecovirus and are therefore ideal targets for the future development of pan-sarbecovirus antibody-based drugs.

## Methods

### Isolation of human peripheral blood mononuclear cells

SARS-CoV-2 convalescent individuals, SARS-CoV-1 convalescent individuals and individuals who had been vaccinated against SARS-CoV-2 were recruited on the basis of previous SARS-CoV-2 infection or SARS-CoV-1 infection at Beijing Youan and Ditan hospitals. Relevant experiments regarding SARS-CoV-2 convalescent individuals and vaccinated individuals were approved by the Beijing Youan Hospital Research Ethics Committee (ethics committee archiving no. LL-2020-010-K). Relevant experiments regarding SARS-CoV-1 convalescent individuals were approved by the Beijing Ditan Hospital Capital Medical University (ethics committee archiving no. LL-2021-024-02). All participants provided written informed consent for the collection of information, and for their clinical samples to be stored and used for research. It was agreed that data generated from the research were to be published. Detailed information on SARS-CoV-2 convalescent individuals and vaccinated individuals has been published previously^11^. In brief, blood samples from short-term convalescent individuals were obtained at day 62 on average after the onset of symptoms. Blood samples from long-term convalescent individuals were obtained at day 371 on average after the onset of symptoms. No vaccination was received before blood collection. Blood samples from individuals who had been vaccinated against SARS-CoV-2 were obtained two weeks after complete vaccination of ZF2001 (RBD-subunit vaccine). For SARS-CoV-1 convalescent individuals who received SARS-CoV-2 vaccines (average age 58, *n* = 21), all recruited participants were previously identified for SARS-CoV-1 infection in 2003, and received a two-dose vaccination of CoronaVac and a booster dose of ZF2001 with a 180-day interval. Blood samples (20 ml) from the SARS-CoV-1 convalescent individuals who were vaccinated against SARS-CoV-2 were obtained two weeks after the booster shot. Three healthy vaccinated donors (average age 25) were also included to serve as negative control for FACS gating. Peripheral blood mononuclear cells (PBMCs) were separated from whole-blood samples based on the detailed protocol described previously^11^. In brief, blood samples were first diluted with 2% fetal bovine serum (FBS) (Gibco) in phosphate buffered saline (PBS) (Invitrogen) and subjected to Ficoll (Cytiva) gradient centrifugation. After red blood cell lysis and washing steps, PBMCs were resuspended with 2% FBS in PBS for downstream B cell isolation or 10% dimethyl sulfoxide (Sigma-Aldrich) in FBS for further preservation.

### Antigen-specific B cell sorting and sequencing

Starting with freshly isolated or thawed PBMCs, B cells were enriched by positive selection using a CD19^+^ B cell isolation kit according to the manufacturer’s instructions (STEMCELL). The enriched B cells were stained in FACS buffer (1× PBS, 2% FBS, 1 mM EDTA) with the following anti-human antibodies and antigens: For every 10^6^ cells, 3 μl FITC anti-CD19 antibody (Biolegend, 392508), 3 μl FITC anti-CD20 antibody (Biolegend, 302304), 3.5 μl Brilliant Violet 421 anti-CD27 antibody (Biolegend, 302824), 3 μl PE/Cyanine7 anti-IgM(Biolegend, 314532), and fluorophore-labelled RBD and ovalbumin (Ova) for 30 min on ice. Cells were stained with 5 μl 7-AAD (eBioscience, 00-6993-50) for 10 min before sorting. Biotinylated RBD of SARS-CoV-1 (Sino Biological, 40634-V27H-B) or SARS-CoV-2 (Sino Biological, 40592-V27H-B) were multimerized with fluorescently labelled streptavidin (SA) for 1 h at 4 °C. RBD was mixed with SA-PE (Biolegend, 405204) and SA-APC (Biolegend, 405207) at a 4:1 molar ratio. For every 10^6^ cells, 6 ng SA was used to stain. Single CD19 or CD20^+^ CD27^+^IgM^−^Ova^−^RBD-PE^+^RBD-APC^+^ live B cells were sorted on an Astrios EQ (BeckMan Coulter) into PBS containing 30% FBS (Supplementary Data 2). FACS sorting was controlled by Summit 6.0 (Beckman Coulter). FACS data analyses were done by FlowJo v.10.8. Cells obtained after FACS were sent for 5′-mRNA and V(D)J library preparation as previously described^11^, which were further submitted for Illumina sequencing on a Hiseq 2500 platform, with the 26×91 paired-end reading mode.

### V(D)J sequence data analysis

The raw FASTQ files were processed by Cell Ranger (v.6.1.1) pipeline using GRCh38 reference. Sequences were generated using ‘cellranger multi’ or ‘cellranger vdj’ with default parameters. Antibody sequences were processed by IMGT/DomainGapAlign (v.4.10.2) to obtain the annotations of V(D)J, regions of complementarity determining regions (CDRs), and the mutation frequency^49,50^. The mutation count divided by the length of the V gene peptide is defined as the amino acid mutation rate of the V gene.

### Recombinant antibody production

Paired immunoglobulin heavy and light chain genes obtained from 10X Genomics V(D)J sequencing and analysis were submitted to recombinant monoclonal antibody synthesis. In brief, heavy and light genes were cloned into expression vectors, respectively, based on Gibson assembly, and subsequently co-transfected into HEK293F cells (Thermo Fisher Scientific, R79007). The secreted monoclonal antibodies from cultured cells were purified by protein A affinity chromatography. The specificities of these antibodies were determined by ELISA.

### ELISA

ELISA plates were coated with RBD (SARS-CoV-2 wild type, SARS-CoV-2 Omicron, SARS-CoV-1 RBD, Sino Biological) at 0.03 μg ml^−1^ and 1 μg ml^−1^ in PBS at 4 °C overnight. After standard washing and blocking, 100 μl of 1 μg ml^−1^ antibodies were added to each well. After a 2-h incubation at room temperature, plates were washed and incubated with 0.08 μg ml^−1^ goat anti-human IgG (H+L)/HRP (Jackson, 109-035-003) for 1 h incubation at room temperature. Tetramethylbenzidine (TMB) (Solarbio) was then added, and the reaction was stopped by adding H_2_SO_4_. Optical density at 450 nm (OD_450_) was measured by an ELISA microplate reader. An antibody is defined as ELISA-positive when the OD_450_ (1 μg ml^−1^ RBD) is three times larger than the negative control, which uses an H7N9-specific human IgG1 antibody (HG1K, Sino Biological).

### Pseudovirus neutralization assay

A pseudovirus neutralization assay was performed to evaluate the neutralizing ability of antibodies. The detailed process has been previously described^12^. In brief, serially diluted antibodies were first incubated with pseudotyped virus for 1 h, and the mixture was then incubated with Huh-7 cells. After a 24-h incubation in an incubator at 37 °C, cells were collected and lysed with luciferase substrate (PerkinElmer), then underwent luminescence intensity measurement by a microplate reader. IC_50_ was determined by a four-parameter non-linear regression model using PRISM (v.9.0.1). Omicron pseudovirus contains the following mutations: A67V, H69del, V70del, T95I, G142D, V143del, Y144del, Y145del, N211del, L212I, ins214EPE, G339D, S371L, S373P, S375F, K417N, N440K, G446S, S477N, T478K, E484A, Q493R, G496S, Q498R, N501Y, Y505H, T547K, D614G, H655Y, N679K, P681H, N764K, D796Y, N856K, Q954H, N969K and L981F.

### Biolayer interferometry

Biolayer interferometry (BLI) assays were conducted on an Octet R8 Protein Analysis System (ForteBio) following the manufacturer’s instructions. In brief, after baseline calibration, Protein A biosensors (ForteBio) were immersed with antibodies to capture the antibody, then sensors were immersed in PBS with 0.05% Tween-20 to the baseline. After association with different concentrations of RBD of SARS-CoV-2 variants (Omicron RBD: 40592-V08H85), disassociation was conducted. Data were recorded using Octet BLI Discovery (12.2) and analysed using Octet BLI Analysis (12.2).

### Construction of RBD deep mutational scanning library

The yeast display RBD mutant libraries used here were constructed as described previously^12^, on the basis of the spike RBD from SARS-CoV-2 (NCBI GenBank: MN908947, residues N331–T531) with the modification that instead of a 16-nucleotide barcode (N16), a unique 26-nucleotide (N26), barcode was appended to each RBD variant as an identifier, to decrease sequencing cost by eliminating the use of PhiX. In brief, three rounds of mutagenesis PCR were performed with designed and synthesized mutagenetic primer pools; to support our conclusions, we constructed two RBD mutant libraries independently. RBD mutant libraries were then cloned into the pETcon 2649 vector and the assembled products were electroporated into electrocompetent DH10B cells to enlarge the plasmid yield. Plasmid extracted form *Escherichia coli* were transformed into the EBY100 strain of *Saccharomyces cerevisiae* using the method described in a previous report^51^. Transformed yeast populations were screened on SD-CAA selective plate and further cultured in SD-CAA liquid medium at a large scale. The resulted yeast libraries were flash-frozen by liquid nitrogen and preserved at −80 °C.

### PacBio library preparation, sequencing and analysis

The correspondence of RBD gene sequence in mutant library and N26 barcode was obtained by PacBio sequencing. First, the bacterially extracted plasmid pools were digested by NotI restriction enzyme and purified by agarose gel electrophoresis, then SMRTbell ligation was performed. Four RBD mutant libraries were sequenced in one SMRT cell on a PacBio Sequel ll platform. PacBio SMRT sequencing subreads were converted to HiFi ccs reads with pbccs, and then processed with a slightly modified version of the script previously described^12^ to generate the barcode-variant dictionary. To reduce noise, variants that contained stop codons or that were supported by only one ccs read were removed from the dictionary and ignored during further analysis.

### MACS-based profiling of escape mutations

ACE2-binding mutants were sorted using magnetic beads to eliminate non-functional RBD variants. In brief, the biotin binder beads (Thermo Fisher Scientific) were washed and prepared as per the manufacturer’s instructions and incubated with biotinylated ACE2 protein (Sino Biological) at room temperature with mild rotation. The ACE2-bound beads were washed twice and resuspended with 0.1% BSA buffer (PBS supplemented with 0.1% bovine serum albumin), ready for ACE2 positive selection. Transformed yeast libraries were inoculated into SD-CAA and grown at 30 °C with shaking for 16–18 h, then back-diluted into SG-CAA at 23 °C with shaking to induce RBD surface expression. Yeasts were collected and washed twice with 0.1% BSA buffer and incubated with the aforementioned ACE2-bound beads at room temperature for 30 min with mild rotating. Then, the bead-bound cells were washed, resuspended with SD-CAA medium and grown at 30 °C with shaking. After overnight growth, the bead-unbound yeasts were separated with a magnet and cultured on a large scale. The above ACE2-positive selected yeast libraries were preserved at −80 °C in aliquots as a seed bank for antibody escape mapping.

One aliquot of the ACE2-positive selected RBD library was thawed and inoculated into SD-CAA, then grown at 30 °C with shaking for 16–18 h. 120 OD units were back-diluted into SG-CAA medium and induced for RBD surface expression. Two rounds of sequential negative selection to sort yeast cells that escape Protein A conjugated antibody binding were performed according to the manufacturer’s protocol. In brief, Protein A magnetic beads (Thermo Fisher Scientific) were washed and resuspended in PBST (PBS with 0.02% Tween-20). Then beads were incubated with neutralizing antibody and rotated at room temperature for 30 min. The antibody-conjugated beads were washed and resuspended in PBST. Induced yeast libraries were washed and incubated with antibody-conjugated beads for 30 min at room temperature with agitation. The supernatant was separated and underwent a second round of negative selection to ensure full depletion of antibody-binding yeast.

To eliminate yeast that did not express RBD, MYC-tag-based RBD positive selection was conducted according to the manufacturer’s protocol. First, anti-c-Myc magnetic beads (Thermo Fisher Scientific) were washed and resuspended with 1× TBST (TBS with Tween-20), then the prepared beads were incubated for 30 min with the antibody-escaping yeasts after two rounds of negative selection. Yeasts bound by anti-c-Myc magnetic beads were washed with 1× TBST and grown overnight in SD-CAA to expand the yeast population before plasmid extraction.

Overnight cultures of MACS-sorted antibody-escaped and ACE2-preselected yeast populations were passed on to a yeast plasmid extraction kit (Zymo Research). PCRs were performed to amplify the N26 barcode sequences as previously described^13^. The PCR products were purified with 0.9X Ampure XP beads (Beckman Coulter) and submitted to 75-bp single-end Illumina Nextseq 500 sequencing.

### Processing of deep mutational scanning data

Raw single-end Illumina sequencing reads were trimmed and aligned to the reference barcode-variant dictionary generated as described above to get the count of each variant with the dms_variants Python package (v.0.8.9). For libraries with N26 barcodes, we slightly modified the illuminabarcodeparser class of this package to tolerate one low sequencing quality base in the barcode region. The escape score of variant X is defined as *F*×(*n*_X,ab_/*N*_ab_)/(*n*_X,ref_/*N*_ref_), in which *n*_X,ab_ and *n*_X,ref_ are the number of detected barcodes for variant X, and *N*_ab_ and *N*_ref_ are the total number of barcodes in the antibody-selected (ab) library and the reference (ref) library, respectively, as described previously^12^. Different to FACS experiments, as we couldn’t measure the number of cells retained after MACS selection precisely, here *F* is considered as a scaling factor to transform raw escape fraction ratios to the 0–1 range, and is calculated from the first and 99th percentiles of raw escape fraction ratios. Scores less than the first percentile or larger than the 99th percentile are considered to be outliers and set to zero or one, respectively. For each experiment, barcodes detected by fewer than 6 reads in the reference library were removed to reduce the effect of sampling noise, and variants with ACE2 binding below −2.35 or RBD expression below −1 were removed as previously described^12^. Finally, we built global epistasis models with the dms_variants package for each library to estimate single mutation escape scores, using the Python scripts provided in a previous report^16^. To reduce experimental noise, a site was retained for further analysis only if its total escape score was at least 0.01, and at least 3 times greater than the median score of all sites. For antibodies measured by two independent experiments, only sites that passed the filter in both experiments were retained. Logo plots in Figs. 2, 3, Extended Data Fig. 2 and Supplementary Data 1 are generated by the Python package logomaker (v.0.8).

### Antibody clustering

Antibody clustering and epitope group identification were performed on the basis of the *N*×*M* escape score matrix, in which *N* is the number of antibodies that pass the quality controlling filters, and *M* is the number of informative sites on the SARS-CoV-2 RBD. Each entry of the matrix *A*_nm_ refers to the total escape score of all kinds of mutations on site *m* of antibody *n*. The dissimilarity between two antibodies is defined on the basis of the Pearson’s correlation coefficient of their escape score vectors; that is, D_*ij*_ = 1 − Corr(**A**_*i*_, **A**_*j*_), in which Corr(**A**_*i*_, **A**_*j*_) = **x**_*i*_**x**_*j*_/|**x**_*i*_||**x**_*j*_| and vector **x**_*i*_ = **A**_*i*_ − Mean(**A**_*i*_). Sites with at least six escaped antibodies (site escape score > 1) were considered informative and selected for dimensionality reduction and clustering. We used the R function cmdscale to convert the cleaned escape matrix into an N×6 feature matrix by multidimensional scaling (MDS) with the dissimilarity metric described above, followed by unsupervised *k*-medoids clustering within this 6-dimensional antibody feature space, using the pam function of the R package cluster (v.2.1.1). Finally, two-dimensional *t*-SNE embeddings were generated with the Rtsne package (v.0.15) for visualization. Two-dimensional *t*-SNE plots are generated by ggplot2 (v.3.3.3), and heat maps are generated by the ComplexHeatmap package (v.2.6.2).

### Reporting summary

Further information on research design is available in the Nature Research Reporting Summary linked to this paper.

## Online content

Any methods, additional references, Nature Research reporting summaries, source data, extended data, supplementary information, acknowledgements, peer review information; details of author contributions and competing interests; and statements of data and code availability are available at 10.1038/s41586-021-04385-3.

## Supplementary information

Supplementary Table 1 Summarized information of all 247 neutralizing antibodies, including their sources, epitope groups, pseudovirus neutralizing IC_50_ for D614G, SARS-CoV, Beta and Omicron variants, and sequences of heavy and light chains.

Reporting Summary

Supplementary Data 1 Escaping mutation profiles of 247 SARS-CoV-2 neutralizing antibodies of 6 epitope groups. For each site, the height of each amino acid represents its mutation escape score. Sites mutated frequently in Omicron variant are highlighted.

Supplementary Data 2 FACS strategy to isolate SARS-CoV-2 RBD and SARS-CoV-1 RBD double-positive B cell for single-cell VDJ sequencing. The target cell population of each step is labelled in the figure.

## Data Availability

Processed escape maps for neutralizing antibodies are available in Supplementary Data 1 (as figures) or at https://github.com/sunneyxielab/SARS-CoV-2-RBD-Abs-HTDMS (as mutation escape score data). Raw Illumina and PacBio sequencing data are available through the NCBI Sequence Read Archive BioProject (accession number PRJNA787091). We used vdj_GRCh38_alts_ensembl-5.0.0 as the reference for V(D)J alignment, which can be obtained from https://support.10xgenomics.com/single-cell-vdj/software/downloads/latest. IMGT/DomainGapAlign is based on the built-in latest IMGT antibody database, and we left the ‘Species’ parameter as ‘*Homo sapiens*’ and kept the others as default. FACS-based deep mutational scanning datasets can be downloaded from https://media.githubusercontent.com/media/jbloomlab/SARS2_RBD_Ab_escape_maps/main/processed_data/escape_data.csv. Processed data from this study have also been added to this repository.

## Code availability

Scripts for analysing SARS-CoV-2 escaping mutation profile data and for reproducing figures in this paper are available at https://github.com/sunneyxielab/SARS-CoV-2-RBD-Abs-HTDMS.
