# Supplementary material for: Omicron escapes the majority of existing SARS-CoV-2 neutralizing antibodies
Source: Nature. 2021 Dec 23;602(7898):657–63. doi: 10.1038/s41586-021-04385-3 (PMC8866119; doi:10.1038/s41586-021-04385-3)
Supplement: Supplementary file 2 — Reporting Summary [file 41586_2021_4385_MOESM2_ESM.pdf]

nature portfolio

Corresponding author(s): Yunlong Cao, Xiangxi Wang, Junyu Xiao, Youchun Wang, and Xiaoliang Sunney Xie

Last updated by author(s): Dec 22, 2021

Reporting Summary

Nature Portfolio wishes to improve the reproducibility of the work that we publish. This form provides structure for consistency and transparency in reporting. For further information on Nature Portfolio policies, see our Editorial Policies and the Editorial Policy Checklist.

Statistics

For all statistical analyses, confirm that the following items are present in the figure legend, table legend, main text, or Methods section.

n/a	Confirmed	
☐	☒	The exact sample size (*n*) for each experimental group/condition, given as a discrete number and unit of measurement
☐	☒	A statement on whether measurements were taken from distinct samples or whether the same sample was measured repeatedly
☒	☐	The statistical test(s) used AND whether they are one- or two-sided<br>*Only common tests should be described solely by name; describe more complex techniques in the Methods section.*
☒	☐	A description of all covariates tested
☒	☐	A description of any assumptions or corrections, such as tests of normality and adjustment for multiple comparisons
☐	☒	A full description of the statistical parameters including central tendency (e.g. means) or other basic estimates (e.g. regression coefficient) AND variation (e.g. standard deviation) or associated estimates of uncertainty (e.g. confidence intervals)
☒	☐	For null hypothesis testing, the test statistic (e.g. *F*, *t*, *r*) with confidence intervals, effect sizes, degrees of freedom and *P* value noted<br>*Give P values as exact values whenever suitable.*
☒	☐	For Bayesian analysis, information on the choice of priors and Markov chain Monte Carlo settings
☒	☐	For hierarchical and complex designs, identification of the appropriate level for tests and full reporting of outcomes
☐	☒	Estimates of effect sizes (e.g. Cohen's *d*, Pearson's *r*), indicating how they were calculated

Our web collection on statistics for biologists contains articles on many of the points above.

Software and code

Policy information about availability of computer code

Data collection	BLI binding data collections were done by using Octet BLI Discovery 12.2.<br>FACS cell sorting were done by using Summit 6.0 (Beckman Coulter).
Data analysis	Neutralization assays were analyzed using PRISM (versions 9.0.1) as described in Methods.<br>BLI binding data analyses were done by using Octet BLI Analysis 12.2.<br>FACS data were analyzed by FlowJo 10.8.<br>V(D)J sequence data were analyzed using Cell Ranger (v6.1.1) and IMGT/DomainGapAlign (v4.10.2).<br>Illumina barcodes sequencing data from deep mutational scanning experiments were analyzed using custom scripts (https://github.com/sunneyxielab/SARS-CoV-2-RBD-Abs-HTDMS) and package dms_variants (v0.8.9).<br>Logo plots were generated by Python package logomaker (version 0.8).<br>For unsupervised clustering, we utilized R function cmdscale to convert the cleaned escape matrix into an N×6 feature matrix by multidimensional scaling (MDS) with the dissimilarity metric, followed by unsupervised k-medoids clustering within this 6-dimensional antibody feature space, using pam function of R package cluster (version 2.1.1). Two-dimensional t-Distributed Stochastic Neighbor Embedding (tSNE) embeddings were generated with Rtsne package (version 0.15) for visualization. 2D t-SNE plots are generated by ggplot2 (version 3.3.3), and heatmaps are generated by ComplexHeatmap package (version 2.6.2).

For manuscripts utilizing custom algorithms or software that are central to the research but not yet described in published literature, software must be made available to editors and reviewers. We strongly encourage code deposition in a community repository (e.g. GitHub). See the Nature Portfolio guidelines for submitting code & software for further information.

Data

Policy information about availability of data

All manuscripts must include a data availability statement. This statement should provide the following information, where applicable:

- Accession codes, unique identifiers, or web links for publicly available datasets
- A description of any restrictions on data availability
- For clinical datasets or third party data, please ensure that the statement adheres to our policy

Data availabilityProcessed escape maps for NAbs are available in Supplementary Data 1 (as figures), or at https://github.com/sunneyxielab/SARS-CoV-2-RBD-Abs-HTDMS (as mutation escape score data). Raw Illumina and PacBio sequencing data are available on NCBI Sequence Read Archive BioProject PRJNA787091. We used vdj_GRCh38_alts_ensembl-5.0.0 as the reference of V(D)J alignment, which can be obtained from https://support.10xgenomics.com/single-cell-vdj/software/downloads/latest. IMGT/DomainGapAlign is based on the built-in lastest IMGT antibody database, and we let the "Species" parameter as "Homo sapiens" while kept the others as default. FACS-based deep mutational scanning datasets could be downloaded from https://media.githubusercontent.com/media/jbloomlab/SARS2_RBD_Ab_escape_maps/main/processed_data/escape_data.csv. Processed data of this study has been added to this repository as well.

Field-specific reporting

Please select the one below that is the best fit for your research. If you are not sure, read the appropriate sections before making your selection.

☒ Life sciences ☐ Behavioural & social sciences ☐ Ecological, evolutionary & environmental sciences

For a reference copy of the document with all sections, see nature.com/documents/nr-reporting-summary-flat.pdf

Life sciences study design

All studies must disclose on these points even when the disclosure is negative.

Sample size	A total of 247 neutralizing antibodies were characterized in the manuscript. No sample size calculation was performed. The sample size of this study is sufficient to obtain sufficient antibodies in each epitope group.
Data exclusions	A total of 271 NAbs were initially planned for yeast-display , and 23 NAbs failed due to technical errors and could not give any meaningful mutation data.
Replication	Experimental assays were performed in biological duplicate or triplicate according to or exceeding standards in the field. Specifically, we perform MACS-based mutation screening using two independently synthesized mutant libraries. We conducted all neutralization and ELISA assays in biological duplicates or triplicates. All replicates for neutralization and binding assays are successful.
Randomization	Randomization was not required since we were applying a uniform set of measurements across the panel of monoclonal antibodies
Blinding	Blinding was not required since we were applying a uniform set of measurements across the panel of monoclonal antibodies

Reporting for specific materials, systems and methods

We require information from authors about some types of materials, experimental systems and methods used in many studies. Here, indicate whether each material, system or method listed is relevant to your study. If you are not sure if a list item applies to your research, read the appropriate section before selecting a response.

Materials & experimental systems

n/a	Involved in the study	
☐	☒	Antibodies
☐	☒	Eukaryotic cell lines
☒	☐	Palaeontology and archaeology
☒	☐	Animals and other organisms
☐	☒	Human research participants
☒	☐	Clinical data
☒	☐	Dual use research of concern

Methods

n/a	Involved in the study	
☒	☐	ChIP-seq
☐	☒	Flow cytometry
☒	☐	MRI-based neuroimaging

Antibodies

Antibodies used	ELISA anibody detection: 109-035-003, Peroxidase-AffiniPure Goat Anti-Human IgG (H+L) , Jackson<br>Negative control H7N9 human IgG1 antibody: HG1K, Sino Biology Cat #HG1K<br>The enriched B cells were stained with the following anti-human antibodies and antigens: For every 10^6 cells, 3 μL FITC anti-CD19 Antibody (Biolegend, 392508), 3 μL FITC anti-CD20 Antibody (Biolegend, 302304), 3.5 μL Brilliant Violet 421 anti-CD27 Antibody

	(Biolegend, 302824), 3 µL PE/Cyanine7 anti-IgM(Biolegend, 314532), and fluorophore-labelled Receptor-Binding Domain (RBD) and ovalbumin (Ova) for 30 min on ice. Cells were stained with 5 µL 7-AAD (eBioscience, 00-6993-50) for 10 minutes before sorting. All neutralizing antibodies were expressed using HEK293F cell lines with codon-optimized cDNA and human IgG1 constant regions in house. The detailed sequence could be found in Supplementary Table 1 column I and J.
Validation	In this manuscript, we tested 247 anti-RBD SARS-CoV-2 human neutralizing IgG1 antibodies. All neutralizing antibodies were expressed using HEK293F cell lines with codon-optimized cDNA and human IgG1 constant regions. All neutralizing antibodies' species and specificity to RBD were validated by ELISA using goat anti-human IgG (H+L)/HRP. All antibodies neutralization ability was verified by VSV-based pseudovirus assays. Details and sequences for all SARS-CoV-2 neutralizing antibodies evaluated in this study is included in Supplementary Table 1.<br>Reactivity and specificity of the primary antibodies listed above is based on the information on manufacturer's homepages.

Eukaryotic cell lines

Policy information about cell lines

Cell line source(s)	HEK293F for antibody production was received from ThermoFisher (R79007)<br>EBY100 (Yeast) was received from ATCC (ATCCMYA-4941);<br>Huh-7 for pseudovirus assays was received from Japanese Collection of Research Bioresources （JCRB 0403) ;
Authentication	No authentication was performed beyond manufacturer standards;
Mycoplasma contamination	Not tested for mycoplasma contamination;
Commonly misidentified lines<br>(See ICLAC register)	No commonly misidentified cell lines were used in the study.

Human research participants

Policy information about studies involving human research participants

Population characteristics	The detailed information of SARS-CoV-2 convalescents and vaccinees was previously described in Cao et al., Cell Research, 2021, doi:10.1038/s41422-021-00514-9. Briefly, short-term convalescents' blood samples were obtained at day 62 on average after symptoms onset. Long-term convalescents' blood samples were obtained at day 371 on average after symptoms onset. No vaccination was received before blood collection. SARS-CoV-2 vaccinees' blood samples were obtained 2 weeks after complete vaccination of ZF2001 (RBD-subunit vaccine).For vaccinated SARS-CoV-1 convalescents (average age 58, n = 21), all recruited participants were identified for SARS-CoV-1 infection in 2003, and received two-dose vaccination of CoronaVac and a booster dose of ZF2001 with a 180-day-interval. Blood samples of vaccinated SARS-CoV-1 convalescents were obtained 2 weeks after the booster shot. Three Healthy vaccinated donor (average age 25) were also included to serve as negative control for FACS gating.
Recruitment	Patients were recruited on the basis of prior SARS-CoV-2 infection or SARS-CoV-1 infection or SARS-CoV-2 vaccination. The only exclusion criteria used were HIV or other debilitating diseases.
Ethics oversight	Relevant experiments regarding SARS-CoV-2 convalescents and vaccinees were approved by the Beijing Youan Hospital Research Ethics Committee (Ethics committee archiving No. LL-2020-010-K). Relevant experiments regarding SARS-CoV-1 convalescents were approved by the Beijing Ditan Hospital Capital Medical University (Ethics committee archiving No. LL-2021-024-02). Written informed consent was obtained from each participant in accordance with the Declaration of Helsinki. All participants provided written informed consent for the collection of information, and that their clinical samples were stored and used for research. Data generated from the research were agreed to be published.

Note that full information on the approval of the study protocol must also be provided in the manuscript.

Flow Cytometry

Plots

Confirm that:

☒ The axis labels state the marker and fluorochrome used (e.g. CD4-FITC).

☒ The axis scales are clearly visible. Include numbers along axes only for bottom left plot of group (a 'group' is an analysis of identical markers).

☒ All plots are contour plots with outliers or pseudocolor plots.

☒ A numerical value for number of cells or percentage (with statistics) is provided.

Methodology

Sample preparation	Whole blood samples from SARS-CoV-2 convalescents or vaccinees were mixed and subjected to Ficoll (Cytiva, 17-1440-03) gradient centrifugation after 1:1 dilution in PBS+2% FBS. After centrifugation, plasma was collected from upper layer and cells were harvested at the interface, respectively. PBMCs were further prepared through centrifugation, red blood cells lysis (InvitrogenTM eBioscienceTM 1X RBC Lysis Buffer, 00-4333-57) and washing steps. Samples were stored in FBS (Gibco) with 10% DMSO (Sigma) in liquid nitrogen if not used for downstream process immediately. Cryopreserved PBMCs were thawed in DPBS+2% FBS (Stemcell, 07905). On the day of sorting, B cells were enriched using CD19+ B cell isolation kit according to the manufacturer's instructions (STEMCELL, 19054). Biotinylated receptor binding domain (RBD) of SARS (Sino biological, 40634-V27H-B) or SARS-CoV-2 (Sino biological, 40592-V27H-B) were multimerized with fluorescently labeled Streptavidin (SA) for 1 hour at 4°C. RBD was mixed with SA-PE (Biolegend, 405204) and SA-APC (Biolegend, 405207) at a 4:1 molar ratio. For every 106 cells, 6 ng SA was used to stain.
Instrument	Astrios EQ (BeckMan Coulter)
Software	Summit 6.0 (Beckman Coulter) for cell sorting; FlowJo 10.8 for data analysis.
Cell population abundance	Memory B cell purity post-sorting is over 90% as measured by 10x sequencing.
Gating strategy	Single CD19 or CD20+, CD27+, IgM-, Ova-, RBD-PE+, RBD-APC+, live B cells were sorted on an Astrios EQ (BeckMan Coulter) into PBS containing 30% FBS. The detailed FSC/SSC gating scheme is showed in Supplementary Data 2. Gates are drown to define positive cells on the basis of unvaccinated healthy donor control.

☒ Tick this box to confirm that a figure exemplifying the gating strategy is provided in the Supplementary Information.
