# Supplementary material for: Omicron escapes the majority of existing SARS-CoV-2 neutralizing antibodies
Source: Nature. 2021 Dec 23;602(7898):657–63. doi: 10.1038/s41586-021-04385-3 (PMC8866119; doi:10.1038/s41586-021-04385-3)
Supplement: Supplementary file 3 — Escaping mutation profiles of 247 SARS-CoV-2 neutralizing antibodies of 6 epitope groups. For each site, the height of each amino acid represents its mutation escape score. Sites mutated frequently in Omicron variant are highlighted. [file 41586_2021_4385_MOESM3_ESM.pdf]

Escape maps of Epitope Group A antibodies

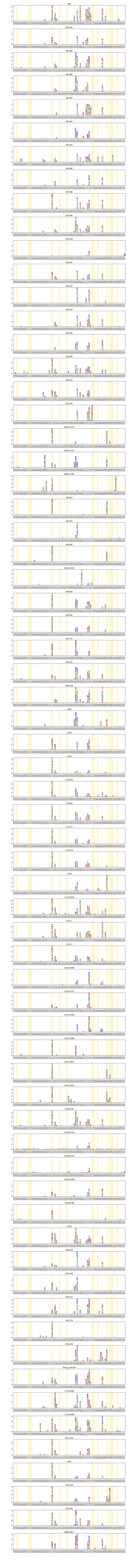

Escape maps of Epitope Group B antibodies

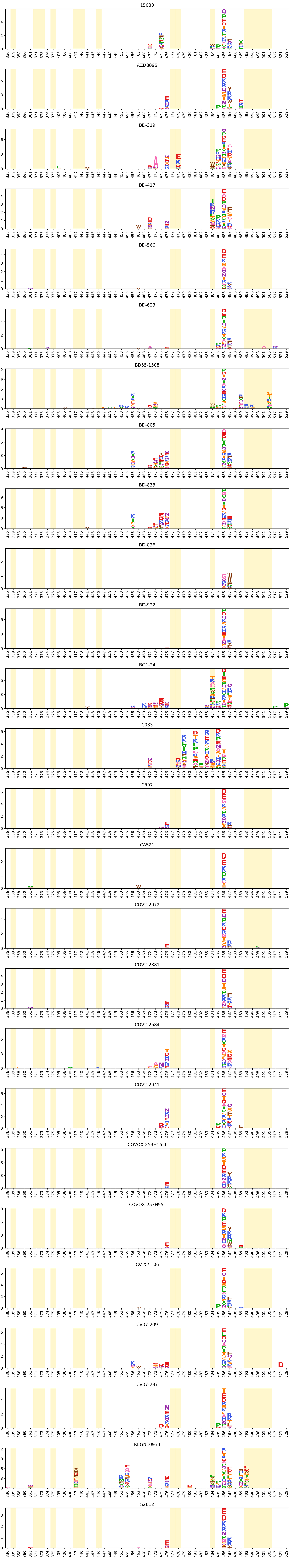

Escape maps of Epitope Group C antibodies

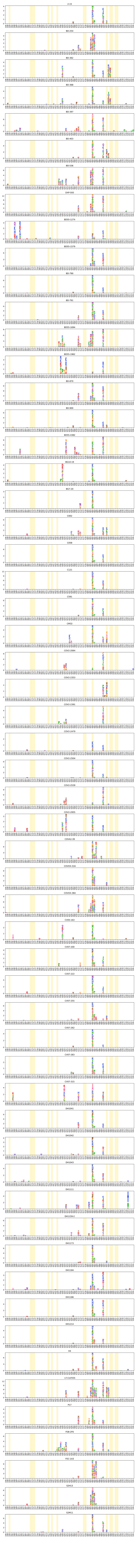

Escape maps of Epitope Group D antibodies

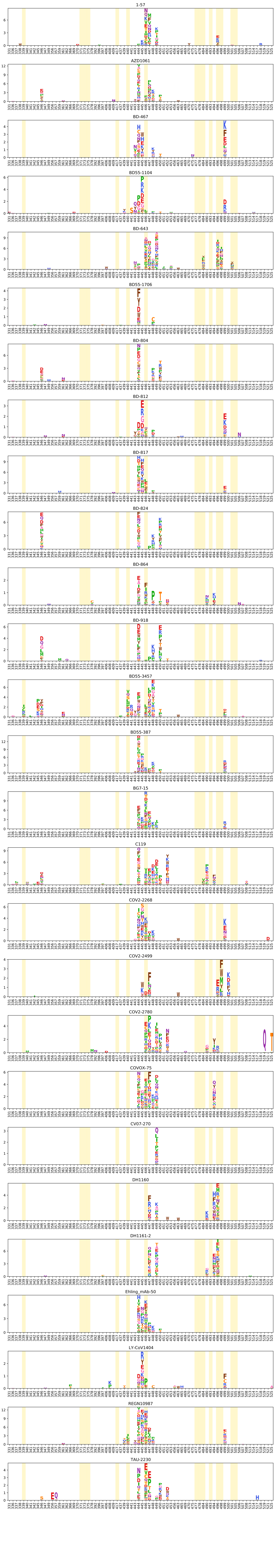

Escape maps of Epitope Group E antibodies

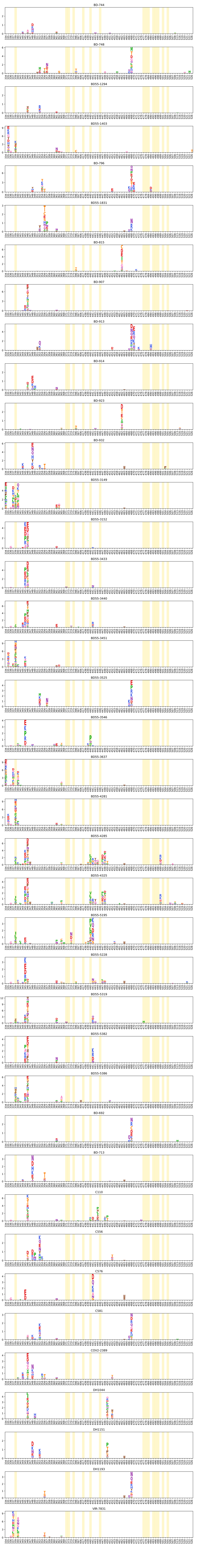

Escape maps of Epitope Group F antibodies

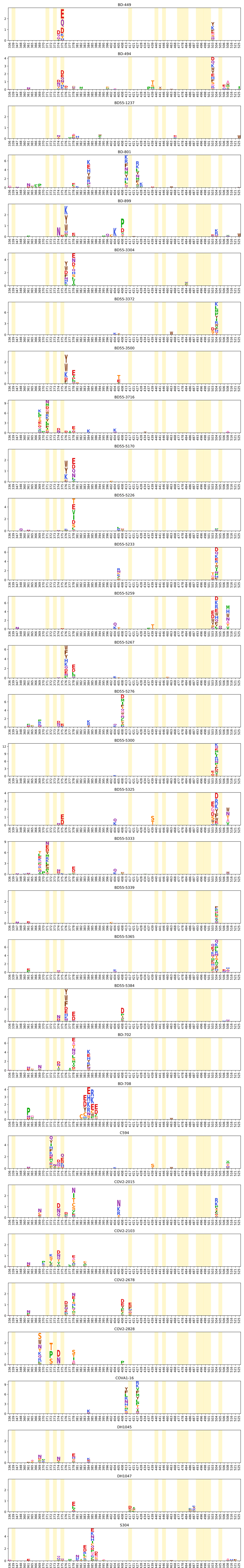

Supplementary Data 1 Escape maps of 247 SARS-CoV-2 RBD antibodies of 6 epitope groups. Page 1-6 correspond to antibodies of epitope group A-F, respectively. For each site, the height of each amino acid residue represents its mutation escape score. Sites mutated frequently in Omicron variant are highlighted. Residues are colored according to their functional groups (*dmslogo_funcgroup* option of *logomaker* Python package).
