# Supplementary material for: Omicron escapes the majority of existing SARS-CoV-2 neutralizing antibodies
Source: Nature. 2021 Dec 23;602(7898):657–63. doi: 10.1038/s41586-021-04385-3 (PMC8866119; doi:10.1038/s41586-021-04385-3)
Supplement: Supplementary file 4 — FACS strategy to isolate SARS-CoV-2 RBD and SARS-CoV-1 RBD double-positive B cell for single-cell VDJ sequencing. The target cell population of each step is labelled in the figure. [file 41586_2021_4385_MOESM4_ESM.pdf]

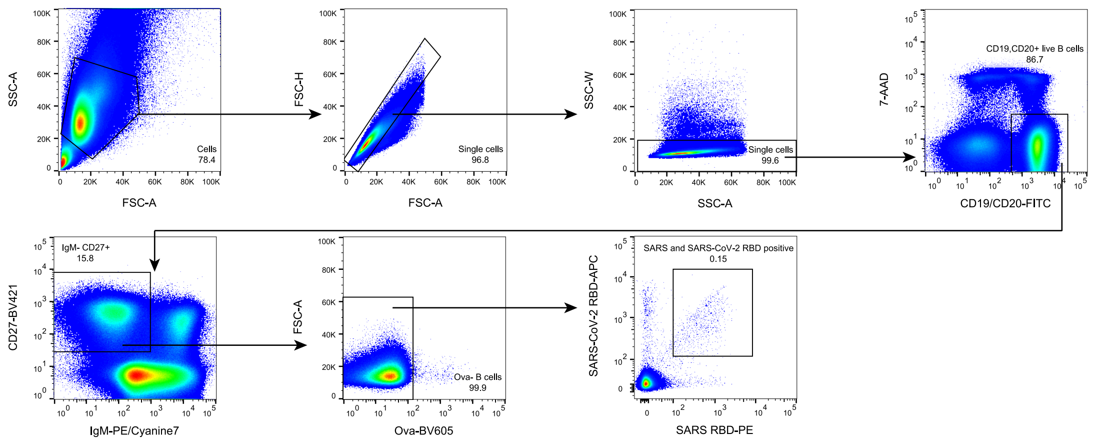

Supplementary Data 2 Flow cytometry gating scheme for antigen-specific memery B cell sorting. Gating was on singlets that were 7-AAD$^-$,CD19 or CD20$^+$, IgM$^-$, CD27$^+$, and Ova$^-$. Sorted cells were SARS RBD$^+$ and SARS-CoV-2 RBD$^+$.
